# Peptide-Mediated Transport Across the Intact Tympanic Membrane Is Intracellular, with the Rate Determined by the Middle Ear Mucosal Epithelium

**DOI:** 10.3390/biom14121632

**Published:** 2024-12-19

**Authors:** Arwa Kurabi, Yuge Xu, Eduardo Chavez, Vivian Khieu, Allen F. Ryan

**Affiliations:** 1 Department of Otolaryngology, UCSD School of Medicine, La Jolla, San Diego, CA 92093-0666, USA; yugex146@gmail.com (Y.X.); chaveze@yahoo.com (E.C.); viviantkhieu@gmail.com (V.K.); afryan@health.ucsd.edu (A.F.R.); 2 Department of Neurosciences, UCSD School of Medicine, La Jolla, San Diego, CA 92093-0666, USA; 3 San Diego VA Healthcare System, San Diego, CA 92093-0666, USA

**Keywords:** tympanic membrane, trans-membrane transport, transcytosis, endocytosis, exocytosis, noninvasive drug delivery

## Abstract

The tympanic membrane forms an impenetrable barrier between the ear canal and the air-filled middle ear, protecting it from fluid, pathogens, and foreign material entry. We previously screened a phage display library and discovered peptides that mediate transport across the intact membrane. The route by which transport occurs is not certain, but possibilities include paracellular transport through loosened intercellular junctions and transcellular transport through the cells that comprise the various tympanic membrane layers. We used confocal imaging to resolve the phage’s path through the membrane. Phages were observed in puncta within the cytoplasm of tympanic membrane cells, with no evidence of phages within junctions between epithelial cells. This result indicates that transport across the membrane is transcellular and within vesicles, consistent with the transcytosis process. The trans-tympanic peptide phages display a wide range of transport efficiencies for unknown reasons. This could include variation in tympanic membrane binding, entry into the membrane, crossing the membrane, or exiting into the middle ear. To address this, we titered phages recovered from within the membrane for phages with differing transport rates. We found that differences in the transport rate were inversely related to their presence within the tympanic membrane. This suggests that differences in the transport rate primarily reflect the efficiency of an exocytotic exit from the mucosal epithelium rather than entry into, or passage across, the membrane.

## 1. Introduction

Diseases of the middle ear (ME) are prevalent. They include otitis media (OM), which affects almost all children and is often treated with systemic antibiotics [1,2]. However, in 10–15% of children, OM turns into a chronic or recurrent condition requiring more aggressive management, such as tympanostomy tubes and/or adenoidectomy [3,4,5]. Another is cholesteatoma, an aggressive growth of keratinized epidermal cells that have invaded the ME. It is often common in individuals with recurring ear infections/OM and eustachian tube dysfunction. It can erode the structures of the middle and even inner ear, causing permanent hearing loss [6]. Cholesteatoma is extirpated surgically, but complete removal can often be difficult, and cholesteatoma frequently recurs due to remnants in the ME.

While systemic antibiotics and tympanostomy tubes are generally effective in chronic OM, they can have significant side effects [7]. Antibiotics may produce gastric distress [8], a potentially serious problem for infants [9]. Because OM is so common, systemic antibiotic therapy can also contribute to antibiotic-resistant bacterial strains throughout the body [10]. In addition, large and infected cholesteatomas are resistant to any type of antimicrobial therapy. Consequently, the complete removal of cholesteatoma is often impossible despite surgery, leaving remnants that lead to future recurrence and repeated surgical removal [11]. Antiproliferative drugs that could inhibit cholesteatoma growth, if used systemically, could adversely affect many other body systems. Local drug delivery restricted to the ME could address the above issues, but drugs cannot penetrate the tympanic membrane (TM), which separates the ear canal from the ME cavity. Tight junctions between cells of the TM epithelia render it impermeable. Breaching the membrane by injection or tympanostomy is currently required for local drug delivery.

Many impermeable epithelia possess mechanisms for macromolecule transit [12,13]. To explore whether the TM possesses such a mechanism, we employed peptide phage display, often used to identify molecules with a desired property [14]. The method employs extensive libraries, where each phage expresses a single, random-sequence peptide on its surface [13]. Phage display requires no prior knowledge of peptide properties: biological function is used for selection, and many peptides with unique properties have been identified via this method [13]. We reasoned that repeated screening of a peptide phage library for the ability to cross the TM might identify clones with trans-TM ability.

Using two biopanning methods, we screened a 12-mer peptide phage library, PhD-12^TM^, from New England Biolabs^®^ [15]. For the first method, we applied the library to the TM of four infected rat MEs, recovered fluid from each ME, and incubated the pooled fluid with *E. coli* to amplify any phage. We then applied the amplified phage to the TM and repeated the process twice. For the second sequential screening method, we applied the original library to the TMs of two infected rat MEs for one hour, rinsed each TM with PBS to remove unbound phage, harvested and homogenized the TMs, and amplified any phage for three rounds to produce a TM-binding library. We then applied this library to the TM, rinsed the TM with PBS plus an acid wash to remove unbound and surface-bound phage, harvested and homogenized the TM, and amplified any phages that were inside the TM for three rounds to produce a TM-penetrating library. Finally, we applied this library to the TM and harvested ME fluid for four rounds to identify TM-transiting phages. Using these two methods, out of 10^10^ peptide phages in the original library, nine entered the ME at much greater rates than wild-type (WT) phages without a peptide, with a range of transport efficiencies. The basis for this variation in the transport rate for different peptides is unclear.

The trans-TM peptides bear no similarity to other known peptides, including cell-penetrating peptides. Transport across the TM is oxygen- and temperature-dependent, suggesting active transport [15]. In an attempt to optimize transport, we extended two of the 12-mer peptides (TMT2 and TMT3) by six random amino acids to create new 18-mer libraries, from which selection yielded one with a much higher rate of transport, TMT3(18)-1 [16]. Using an in vitro assay, we found that trans-TM peptide phage also crosses the human membrane [17]. Finally, inhibitor studies provided evidence that transport across the membrane occurs via micropinocytosis, which is capable of engulfing very large particles, suggesting that transcytosis is the likely mechanism of transport [18,19].

For the current study, we visualized an immunolabeled phage, as it transited the TM by confocal microscopy to explore the transport route and identify the involved TM cells. Also, to investigate the basis for differences in transport rates, we quantified the number of peptide phages within the TM after exposure to the external surface of the phage with widely differing transport rates.

## 2. Materials and Methods

### 2.1. Animals

All experiments were performed in male Sprague Dawley rats (Inotiv) and were performed to NIH standards for the care and use of research animals. Experiments were approved by the Institutional Animal Use and Care Committee of the San Diego Veterans Administration Hospital, protocol A13-022 (approve date 06/07/2022).

### 2.2. Bacteriophage

The Ph.D-12 Phage Display Peptide Library (New England Biolabs^®^) was screened to identify peptides supporting transit across the tympanic membrane. M13 bacteriophages were engineered to display a copy of one of 2.9 × 10^9^ random 12-mer peptides at the free end of each of the five filamentous pIII proteins. Phage were amplified in ER2378 tetracycline-resistant *E. coli*, allowing the blue-white screening for tittering. Once trans-TN phages were identified, they were amplified for individual application to the TM. The M13KE WT phage, which did not bear a peptide, was used as a control.

### 2.3. Infection of the ME and Application of Phage to the TM

Rodent cocktail (2.0 mg/kg xylazine and 40.0 mg/kg ketamine i.m.) was employed for anesthesia. The ME bulla was exposed via a ventral midline neck incision. A 25 g needle was used to make a small opening in the bullar bone, through which 5 × 10^4^ CFUs of nontypeable *Haemophilus influenzae* (NTHi) type 3655 [20] was injected, in 50 μL of BHI media using a 30 g needle. Care was taken to avoid damaging the tympanic membrane, ossicles, or inner ear. Animals survived for 48 h to allow for an ME infection to develop, which was confirmed by TM visualization. Phages were then applied to the external TM surface in vivo for one hour in 10^9^ PFUs (particle forming units) in 10 μL of PBS, ensuring the entire surface of the membrane was covered. For trans-TM rate determination and TM phage content, the phages TMT2 (TLSPKMPGGGYW), TMT3 (SADSTKTTHLTL), and TMT3(18)-1 (SADSTKTTHLTLEGHLFP) were employed.

### 2.4. Confocal Imaging of Trans-TM Phage Within the Tympanic Membrane

For confocal microscopy, TMT3 and WT phages were employed. The animal was sacrificed, the phage solution removed, and the TM washed 3× with PBS to remove any remaining phage solution, then 2× with acid wash to remove phages bound to the TM, followed by 2× with PBS. The bulla was then dissected and opened, the interior TM surfaces were washed as above, and the TM was fixed in cold 4% PFA overnight. The TM was dissected from the annular bone and additionally washed 2× with PBS, and the manubrium of the malleus carefully removed. The membrane was incubated in tissue-penetration and blocking solution (5% Triton X and 10% fetal bovine serum (FBS) in PBS) for one hour at room temperature. The TMs were again washed 2× in PBS and incubated in primary antibody (M13 anti-phage coat protein, rabbit polyclonal antibody, Invitrogen PA1-26758, 1:100) overnight at 4 °C. TMs were washed 2× in PBS and incubated with a secondary antibody (FITC-labeled anti-rabbit, Jackson Immuno, 711-095-152) previously treated with DyLight 405 stain for enhanced visualization (Jackson Immuno 711-475-152) at 1:100 concentration for three hours at room temperature with light protection. TMs were again washed with PBS 2× and stained with DAPI (Novus NPB2-31156, 1:1000, 30 min at room temperature with light protection). Some TMs were also incubated with Texas Red-X Phalloidin (Invitrogen T7471, F-actin staining) at 1:500 concentration for one and one-half hours at room temperature and light protection to stain actin. After incubations, TMs were washed 2× with PBS and mounted with the external surface uppermost using antifade, hard-mount media (Vectashield, VectaLabs H-1400). Control TMs were exposed for 1 h to PBS in vivo and then identically treated.

### 2.5. Confocal Imaging

The localization of the phage was evaluated via fluorescence confocal microscopy. TMs were imaged on a Leica SP8 Confocal with Lightning-adaptive deconvolution or an Olympus FV1000 Confocal with SIM Scanner, and 60× and 100× image stacks were generated for rendering of stained TMs. Image stacks were post-processed using Velocity high-performance software to generate images. Separate portions of the stacks were used to visualize phages in the external epithelium, the middle fibrous layer, and the inner mucosal epithelium. The number of phage puncta in cells where nuclei were observed in the cross-section was quantified using ImageJ to compare the external and mucosal epithelia.

### 2.6. Dynamics of Phage Recovery from the ME During Screening

We evaluated previously unpublished titers of the bacteriophage library used during the original sequential screening as described in Ref. [15]. This included the rounds for selecting phages bound to the external TM surface, recovered from the TM interior, or present in the ME.

### 2.7. Quantitative Analysis of Trans-TM Phage Uptake Within the TM

To evaluate the relationship between trans-TM transport efficiency of peptide phages and their presence within the TM, we chose the low transport rate 12-mer peptide phage TMT2, the high transport rate 12-mer peptide phage TMT3 [15], and the very high transport rate 18-mer peptide phage TMT3(18)-1 [16]. As a control, we used WT phage, which does not bear a peptide. Each peptide phage was applied to the external TM surface (10^9^ PFU in 10 μL of PBS) of six previously infected rats, ensuring that the entire membrane surface was covered for one hour. The phage solution was removed from the external canal, and the surface of the TM was then washed 3× with PBS to remove any phage solution remnant, followed by 2× with 0.2 M glycine acid wash (pH 2.2) to remove any peptide phage bound to the TM surface. The ME bulla was harvested and opened. The interior surface of the TM was washed 3× with PBS and 2× with acid wash. The TM was again washed with PBS 2× on both sides, excised from the annular ring, and homogenized. A homogenate sample was plated onto an *E. coli* culture to titer phage in the sample, and the titers were corrected to reflect all phages within the TM. The results were compared to the recovery of the same phage from the ME lumen after a one-hour application to the external surface of the TM, as previously published [15,19]. Six replicates were averaged to calculate the mean and the standard deviation. The data were compared using ANOVA Kruskal–Wallis using GraphPad Prism 10.

## 3. Results

### 3.1. Bacteriophages Expressing a 12-Mer Trans-TM Peptide Are Observed Within Cells of the TM

**Figure 1:**
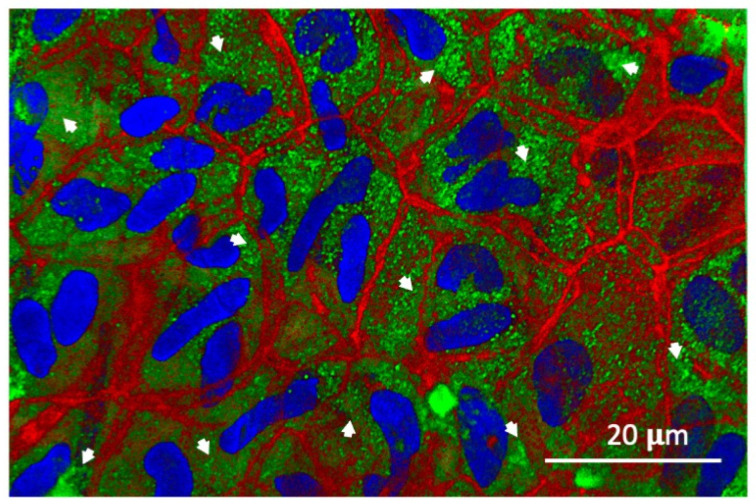
The confocal image of trans-TM peptide phage TMT3 within cells in the external epithelium of the TM. The representative confocal image shows the anti-phage antibody (green fluoresce) as uniformly distributed puncta (white arrows) in the cytoplasm of external epithelial TM cells. No phage staining is present in cell nuclei (DAPI, blue) or in junctions between epithelial cells. The actin network (red) forms a detailed filamentous pattern, contributing to the overall cellular morphology.

External epithelium. The confocal image in Figure 1 illustrates the distribution of TMT3 phage within the external epithelial cells of the TM after a one-hour incubation on the external surface. Phage immunolabeling is visualized as abundant 100–300 nm green puncta. The puncta are clearly intracellular and are restricted to the cell cytoplasm. Phage labeling is present in all epithelial cells, with some variation in the number of puncta per cell. An image analysis with ImageJ revealed that the mean number of puncta per cell was 139.2 (s.d. 43.1, median 110), which across cells was symmetrically distributed. Little or no labeling is present in the junctions between cells. A few clumps of phage, which persisted through PBS and acid washes, are observed on the TM surface. Double nuclei within some cells reflect the dynamic renewal of TM epithelial cells, which continually proliferate in the central zone of the membrane and migrate toward its edges [21].

**Figure 2:**
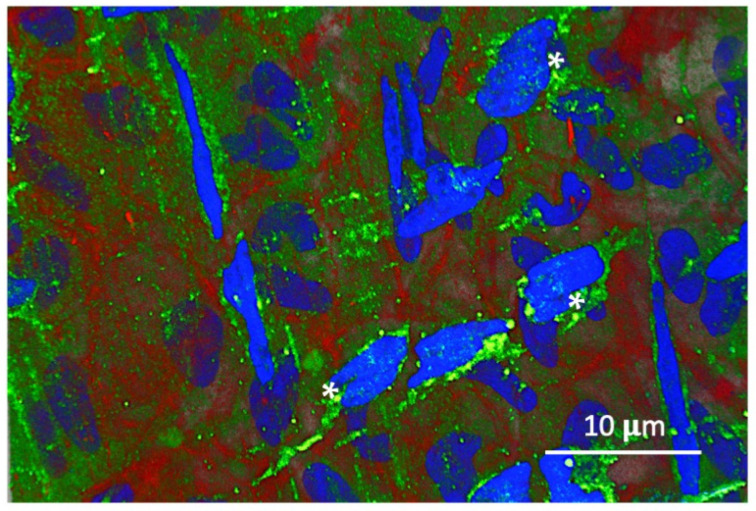
Trans-TM phage TMT3 within the central, fibrous layer of the TM. The phage is present as puncta (stained green) within the cytoplasm of elongated connective tissue cells, where it clusters in the perinuclear region and extracellularly (asterisk). Green puncta are excluded mainly from actin networks (stained red) within and outside cells.

Figure 2 illustrates TMT3 phage within the fibrous internal layer of the TM. As with external TM epithelial cells, an immunolabel is present as green puncta within the cytoplasm of stromal cells but is also present extracellularly. Unlike in external TM epithelial cells, within fibroblasts, they are preferentially located in the perinuclear region. Few puncta are associated with either intracellular or extracellular actin networks.

**Figure 3:**
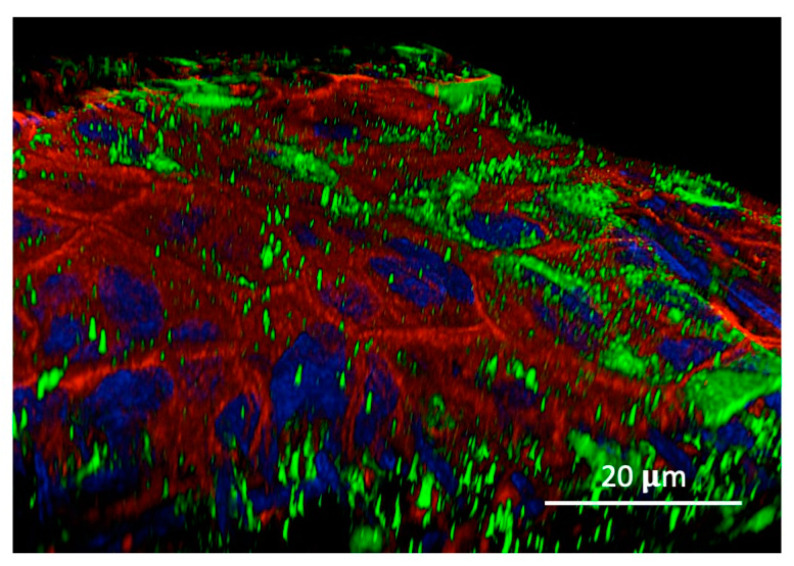
Trans-TM phage TMT3 within cells of the internal mucosal epithelium of the TM. DAPI blue fluorescence highlights the nucleus; red staining: phalloidin to visualize the F-actin. Phage puncta (green) are uniformly distributed in cytoplasm but excluded from nuclei and between cells. Variation in puncta density within different cells is greater than in the external TM epithelium.

**Mucosal epithelium**. Figure 3 shows the distribution of the TMT3 phage within the inner mucosal epithelium of the TM. Again, phage immunolabeling in puncta is restricted to the epithelial cell cytoplasm and is not localized to intercellular junctions. More variability in the number of puncta within different mucosal epithelial cells was observed than in the external TM epithelium. The mean number of puncta per cell was 42.9 (s.d. 44.8), with a median of 23, significantly less than in the external TM epithelial cells (Mann–Whitney Up-Test, p 0.002). The distribution across cells showed a strong right/positive skew with a normality of 8.9 × 10^−7^.

### 3.2. Wild-Type Bacteriophage Not Expressing a Peptide Does Not Enter TM Cells

**Figure 4:**
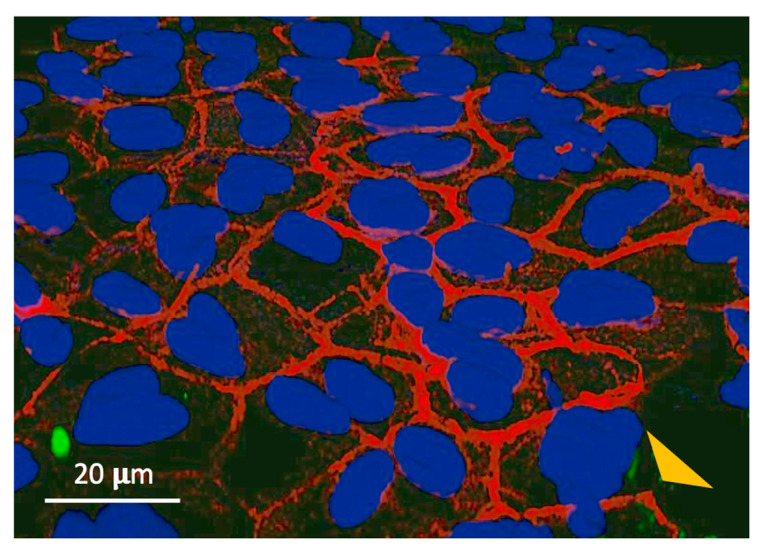
The absence of the control phage (WT) not expressing a peptide within cells of the external epithelium of the TM. The phage alone cannot penetrate the TM. Only M13 phage antibody (green fluorescence) clumps are present on the external TM epithelial surface (yellow arrow). DAPI staining (blue) highlights the cell nuclei, while Phalloidin staining (red) reveals the actin filaments within the cytoskeleton.

Figure 4 shows the immunolabeling of the TM following a one-hour incubation of WT phages not expressing a peptide on the external canal TM surface. No phages were observed inside the epithelial cells or elsewhere within the membrane. Only clumps of phages on the TM surface are present.

**Figure 5:**
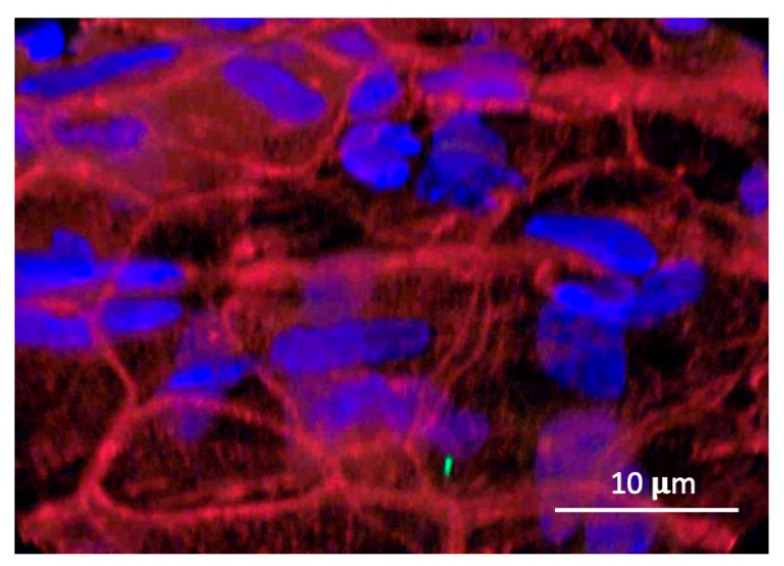
The external epithelium of the TM with no phage applied after immunolabeling for phages. The DAPI-stained nuclei (blue fluorescence) are clearly distinguished from the red actin filaments.

As illustrated in Figure 5, control TMs in which no phage was applied showed no immunolabel (antibody cross-reactivity) within TM cells.

### 3.3. Recovery of Bacteriophage from Within the TM Varies Inversely with Transport Efficiency

**Figure 6:**
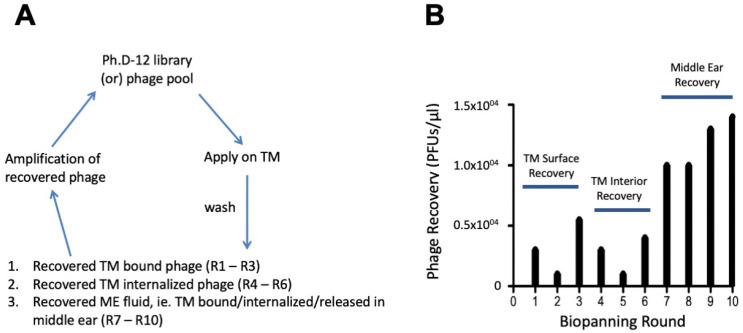
The recovery of phages from the TM during sequential biopanning to discover trans-TM peptides. (**A**). The screening strategy consists of sequential selection for TM binding, TM internalization, and entry into the ME. (**B**). Phage titers are shown from the ten rounds of selection. Three rounds are from selection for TM binding, three from selection for TM internalization, and four from selection for ME entry.

To assess the dynamics of phage entry into the TM, we first assessed the titers obtained in each of the ten selection rounds in the sequential screen for TM-transiting phages, as described above. As shown in Figure 6, phages from the three rounds of library screening for TM binding were high for the first round, low for the second, and higher for the third. A similar result was observed for the three rounds of screening for TM internalization. Only in the final four rounds of screening for recovery from the ME did the phage titer increase sequentially.

**Figure 7:**
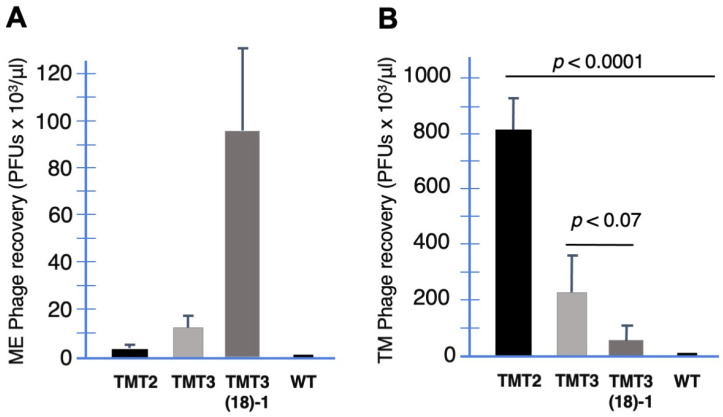
The relationship of trans-TM phage transport efficiency versus recovery from within the TM. (**A**). ME recovery of phage after a one-hour incubation on the external TM surface for peptide phage species with three different levels of trans-TM transport efficiency. (**B**). The recovery of phages from within the TM for each of the identical phage clones, which is inversely related to transport efficiency (**A**). Phage titers (PFUs) are presented as the mean ± standard deviation (SD) derived from six animals per condition.

The above data reflect the behavior of mixtures of phage. To address the role of entry into and presence in the TM for individual phages with different transport rates, we titered phage recovered from the TM after in vivo incubation on its external surface for three peptide phages with very different transport rates. As can be seen in Figure 7, the TM titers of the phage expressing TMT2, with the lowest transport rate, were significantly higher than those of TMT3 (*p* < 0.001), which in turn were higher than that of TMT3 (18)-1 (*p* < 0.0001), which had the highest transport rate. Thus, the number of phages within the TM was inversely related to the transport rate. Control phages (WT) not expressing a peptide were recovered from the TM at negligible levels.

## 4. Discussion

### 4.1. Summary

Confocal imaging of the 12-mer peptide phage TMT3 following application to the outer tympanic membrane surface revealed the presence of phages as ~300 nm puncta, relatively evenly distributed within the cytoplasm of surface epithelial cells. Phalloidin, a particular probe for actin filaments conjugated to fluorescent dye, was used to visualize the actin structures within the tympanic membrane cells. The staining highlighted the organization and filamentous structure of actin in the underlying cellular architecture, enabling a detailed examination of the actin cytoskeleton. The actin filaments appeared as bright, well-defined structures, and all external epithelial cells were labeled. Minimal or no immunoreactivity was observed in the junctions between epithelial cells (Figure 1 and Figure 3). In the fibrous layer of the TM, the label was detected both extracellularly and intracellularly. In stromal cells, the label was predominantly localized to the perinuclear zone. A range of intracellular immunolabeling was also noted, particularly in the mucosal epithelial cells on the inner TM surface (Figure 3).

No labeling within the TM was observed for WT phages not expressing a peptide (Figure 4). During sequential biopanning to identify peptide phages capable of crossing the intact TM, titering confirmed the presence of phages within the TM after each of three sequential applications of a TM-binding phage library to its outer surface (Figure 6). When peptide phages with different rates were applied to the external surface of the TM, transport rates were found to be inversely related to their presence within the TM (Figure 7).

### 4.2. Implications for the Mechanism of Transport

In this study, the absence of phages in the junctions between epithelial cells supports the hypothesis that peptide-mediated transport across the membrane is transcellular, not paracellular. As noted above, this could be mediated by a variety of mechanisms. Peptides could enhance the ability of phages to passively penetrate the membranes of TM cells, followed by diffusion throughout the cell and exit through the membranes on other cell surfaces. Several aspects of our data are inconsistent with this possibility. First, this would presumably result in the movement of phages across lateral epithelial membranes and into the intercellular spaces between epithelial cells, which was not observed. Also, we have previously shown that peptide-mediated trans-TM transport is oxygen dependent and thus active [15].

Another possible mechanism is the movement of individual peptide phages into TM cells via an adaptation of the mechanism that mediates penetration into *E. coli*. This involves interactions between P3 filaments of the phage and TOLA molecules in the bacterium [22], which opens the membrane of *E. coli* and allows for the injection of phage DNA. This would require a compatible protein in TM cell membranes. It would also require a change from DNA injection to internalization of the intact phage since M13 phage cannot replicate in mammalian cells, an unlikely scenario. Moreover, in this case, as with passive penetration of cell membranes, the intracellular labeling of phages would be diffuse, not localized to puncta. When collapsed into a spherical particle, the M13 phage measures ~50 nm in diameter [23]. Even decorated with antibodies, the ~300 nm puncta are too large to represent individual phages.

Finally, phages could be internalized by endocytosis, transported across cells in endosomes, and excreted by exocytosis. This process, known as transcytosis, exists in other polarized cell barriers, can operate in either an apical-to-basal or basal-to-apical direction, and is active. Our observation of phages in large puncta within the cytoplasm of TM cells is consistent with this mechanism. Moreover, trans-TM peptides linked to a DNA template are transported across the TM with the same efficiency as peptides expressed on phages [17]. The equal transport of particles of very different sizes is more consistent with endocytosis than other internalization mechanisms. Finally, we previously found that inhibitors of endocytosis or exocytosis inhibited trans-TM transport of peptide phages, supporting transcytosis as its mechanism [19]. The transport of vesicles through cells can be mediated by actin/myosin interactions or by kinesin or dynein on the endoplasmic reticulum. As we did not see phage puncta associated with actin filaments within cells, the latter seems the more likely mechanism. Colocalization studies using fluorescent markers specific to vesicle membranes could be employed to provide additional evidence for the involvement of vesicular pathways in the transport process.

We noted that the distribution of phage puncta was distinct in cells within the connective layer of the TM. Rather than uniform cytoplasmic distribution, as indicated in epithelial cells (Figure 3 and Figure 5), stromal cells exhibited a perinuclear distribution. The perinuclear region is increasingly recognized as a structure distinct from the cytoplasm [24]. The trafficking of receptors in endosomes to the perinuclear region has been recognized for some time [25]. When trafficked to the perinuclear zone, endosomes often form a perinuclear “cloud” accumulated for receptor inactivation or content degradation [26,27]. While trans-TM peptide phages are clearly internalized into stromal cells, they do not appear to enter the same intracellular transportation network. It can be speculated that they are degraded in the perinuclear recycling region rather than exiting the cell.

We also observed more variation in the number of phage puncta in different cells of the inner mucosal epithelium of the TM. This may reflect specialization between mucosal cells for endo- and exocytosis. Morphological studies of the ME mucosa have previously noted subcategories of ME mucosal epithelial cells, including non-secretory and more than one type of secretory cells [28].

### 4.3. Peptide Phage Entry into the TM During Library Screening

When a “TM binding” library was applied three times to the TM in the initial biopanning study, the phage was recovered from within the TM at a much lower rate in the second application. This could reflect a complex interaction between phage selection and replication efficiency. The initial application could have captured more peptide phages with low TM-entry efficiency, with the resulting library dominated by phages with high replication efficiency. Recovery from the second application would likely have recovered more clones with high TM-entry efficiency but with less selection for high-replication efficiency, resulting in a lower titer. The third application would presumably have involved the most efficient TM-entering and replicating clones, resulting in the highest titer. Another possibility is that the endocytosis of a single TM-binding phage allowed many additional non-TM-entering phages to “piggyback” into the TM. The number accompanying TM-penetrating phages would have been high in the initial application. In the second library application due to a higher proportion of TM-penetrating clones, the number of non-TM-entering phages to piggyback could have been much lower, resulting in somewhat more endocytotic vesicles but fewer phages per vesicle. In the third round, the high proportion of efficient TM-penetrating clones would have dominated TM entry, leading to much higher numbers of vesicles and higher titer. Our data cannot distinguish between these two possibilities. As noted above, colocalization staining with a vesicle marker would be instrumental in distinguishing between these possibilities.

### 4.4. Implications for the Basis of Variation in Peptide-Mediated Transport Rate

Among the peptide phages discovered in our phage library screens, there was a wide range of transport efficiency as reflected in phage recovery from the ME [15]. This could result from limitations anywhere in the transcytosis process, including endocytosis, vesicle transport across TM cells, or exocytosis. The results obtained by titering the TM found that peptide phages with lower transport rates accumulated in the TM at higher levels than those with more efficient transport characteristics. This perhaps counterintuitive result indicates that uptake into the TM is unlikely to be a factor that differentiates peptides conferring different rates to phages, in which case we would have expected to observe a positive relationship between the transport rate and levels within the TM. It is likely that more efficient trans-cellular movement and/or exocytosis differentiates peptides with higher transport rates. Differences in transport across the TM would result in lower levels of phages within mucosal epithelial cells, which we observed. However, the distribution of mucosal phage numbers was highly skewed, and the mucosal cells with the highest TMT3 phage content were comparable to those with the highest numbers in the external epithelium (200–250 puncta/cell). Variations in mucosal epithelial cell phage entry and/or exocytosis could account for different trans-TM transport efficiency.

### 4.5. Implications for the Noninvasive Delivery of Therapies to the ME

As noted above, local delivery of therapies to the ME would have a number of advantages over systemic administration. Other methods for crossing the intact membrane have been devised by others, including penetrants to increase permeability to drugs [29] or driving drug-loaded iron nanoparticles using magnets [30]. As yet, no method has been approved as effective and safe for patients. However, the existence of a mechanism for the active transport of large particles across the intact TM offers an additional opportunity to deliver a diverse array of therapies to the ME without the need to breach the membrane surgically. Because M13 bacteriophage phages are large, this indicates that substantial drug packages, such as nanoparticles or even larger vehicles, can be delivered through the membrane by decoration with appropriate peptides. Large gene therapy vectors could similarly be delivered. The bacteriophages used in this study are viral vectors engineered to encode a transgenic protein for expression in a host organism. While the host was *E. coli*, used to amplify the phage after recovery from the ME, the transport of engineered bacteriophages across the TM provides proof of concept for the noninvasive delivery of other gene therapy vectors to the ME.

## 5. Conclusions

The phage display identified rare peptides that mediate active transport across the intact tympanic membrane. In this study, confocal imaging localized peptide-expressing phages within the cells of the membrane, with no evidence of paracellular transport between tympanic membrane cells, and differences in transport efficiency are linked to peptide-specific exit rates from the membrane. The successful transport of large cargo, nearly 1 μm in length, demonstrates the capacity of these peptides to carry large cargo, with therapeutic potential for the noninvasive delivery of drugs and gene therapy vectors into the middle ear.

## Data Availability

Data are available upon request; please contact the corresponding author with any requests or questions.
